# Early spatial pain contraction and moderate-to-severe knee pain 12 months after total knee arthroplasty: a retrospective cohort study

**DOI:** 10.3389/fpain.2026.1948322

**Published:** 2026-10-12

**Authors:** Jun Liu, Wenheng Chen, Shan Gao

**Affiliations:** Department Joint Surgery, Zhumadian Central Hospital, Zhumadian, China

**Keywords:** pain extent index, pain trajectory, postsurgical pain, subacute recovery, total knee arthroplasty

## Abstract

**Background:**

While total knee arthroplasty (TKA) generally provides excellent pain relief, a substantial minority of patients develop persistent postsurgical pain. This study investigated whether the early postoperative contraction rate of whole-body spatial pain extent was associated with moderate-to-severe knee pain 12 months after TKA.

**Methods:**

In this retrospective cohort study of patients undergoing primary unilateral TKA, participants completed whole-body pain drawings at 2 weeks, 6 weeks, and 3 months postoperatively. The Pain Extent Index (PEI) was calculated using a semiautomated image-processing workflow. The primary exposure was the patient-specific rate of PEI change between week 2 and month 3, determined via linear mixed-effects modeling. The primary outcome was moderate-to-severe operated-knee pain at 12 months. Logistic regression evaluated this association, adjusting for baseline covariates and static pain measures.

**Results:**

At 12 months, 92 patients (14.7%) reported moderate-to-severe knee pain. Slower spatial pain contraction was significantly associated with higher odds of 12-month pain (adjusted odds ratio per 1-SD slower contraction, 1.42; 95% CI, 1.03–1.95). This association remained significant even after adjusting for baseline risk factors and static pain intensity and extent at both week 2 and month 3. Slower contraction was also correlated with poorer 12-month joint-related functional outcomes, including lower KOOS-JR scores (*P* < 0.001).

**Conclusion:**

Slower contraction of spatial pain extent during the subacute recovery phase was associated with moderate-to-severe knee pain and inferior functional outcomes 1 year after TKA.

## Introduction

Total knee arthroplasty (TKA) provides substantial pain relief and functional improvement for most patients with end-stage knee osteoarthritis, but recovery is not uniformly successful. Clinically important postsurgical pain persists in a substantial minority and is accompanied by poorer function and quality of life (1). Long-term follow-up also indicates that unfavorable pain and function trajectories can remain distinguishable for years rather than representing only delayed short-term recovery (2). Identifying patients on an adverse path while recovery may still be modifiable is therefore a major clinical priority.

Most prognostic research has concentrated on presurgical or single-time-point characteristics. Higher baseline pain burden, a greater number of painful locations, and self-reported central sensitization have been associated with worse pain 1 year after TKA (3). Mechanical hyperalgesia and neuropathic pain qualities may also carry prognostic information, although their performance has varied across cohorts (4). Psychological factors, particularly catastrophizing and anxiety, are likewise associated with chronic postoperative pain (5). These findings support a biopsychosocial approach to risk assessment, but static predictors cannot show how an individual responds to the surgical insult or whether recovery is progressing during the subacute phase.

Pain after surgery is inherently dynamic. Repeated assessment can identify distinct acute postoperative pain-impact trajectories that are obscured by isolated measurements (6). Nevertheless, most trajectory studies have focused on pain intensity or interference. Whole-body pain drawings capture a different dimension: the anatomical extent of pain around the operated knee, along the limb, and at remote sites. This spatial dimension may reflect both local surgical recovery and broader pain-system involvement, but it is rarely incorporated into postoperative prognostic models. Whether early postoperative contraction of this spatial burden provides prognostic information beyond pain extent and intensity measured at single visits remains unknown.

We therefore quantified the whole-body Pain Extent Index (PEI), and examined whether slower contraction was associated with moderate-to-severe operated-knee pain at 12 months. We hypothesized that slower early spatial contraction would identify an unfavorable recovery process and would be associated with poorer pain and joint-related outcomes.

## Methods

### Study design and participants

This retrospective analysis of data collected prospectively was conducted at Zhumadian Central Hospital between January 2022 and June 2025. The study was structured as a 3-month landmark cohort: exposure was ascertained from pain drawings obtained at 2 weeks, 6 weeks, and 3 months after surgery, and outcomes were assessed after the landmark. The Institutional Research Committee of Zhumadian Central Hospital approved the study; all participants provided written informed consent, and the study conformed to the Declaration of Helsinki.

Eligible patients were aged 40 years or older and underwent primary unilateral TKA for primary knee osteoarthritis. Exclusion criteria were revision, unicompartmental, patellofemoral, or simultaneous bilateral arthroplasty; inflammatory, post-traumatic, or neuropathic arthropathy; previous ipsilateral knee infection; active malignancy; severe cognitive impairment; contralateral knee or hip arthroplasty before the landmark; and index-knee reoperation, death, or severe illness before 3 months.

### Pain assessment and PEI quantification

Patients completed the anterior and posterior whole-body pain drawing within 4 weeks before surgery and at approximately 2 weeks, 6 weeks, 3 months, 6 months, and 12 months after surgery (Supplementary Figure S1). At each assessment, every painful area during the preceding 7 days was shaded, average operated-knee pain was rated on an 11-point numerical rating scale (NRS; 0 = no pain and 10 = worst imaginable pain).

Paper drawings were scanned at 300 dpi; electronic drawings were exported as lossless PNG files from the same template. Investigators processed the images using Python 3.11.9, OpenCV 4.10.0, SimpleITK 2.4.0, and scikit-image 0.24.0. The semiautomated workflow corrected orientation, removed annotations and nonpain marks, aligned anatomical landmarks, applied affine and nonrigid registration, extracted pain markings, created binary masks, and excluded pixels outside the standardized silhouette. Every mask overlay underwent structured quality control; flagged maps were independently reviewed and resolved by consensus.

The PEI was the percentage of pixels within the combined anterior and posterior silhouettes marked as painful. Regional PEIs were calculated for the operated knee, operated lower extremity, contralateral lower extremity, and extra-articular regions.

Reproducibility was evaluated in all three early drawings from a 20% sample of analyzed patients. A second investigator repeated the complete workflow, and the primary investigator repeated it after at least 4 weeks. PEI agreement was assessed using two-way absolute-agreement intraclass correlation coefficients (ICCs), Dice coefficients, mean absolute differences, and Bland-Altman limits of agreement. Paper-versus-electronic analyses evaluated robustness and effect modification.

### Early spatial pain-contraction velocity

The primary exposure was the patient-specific rate of change in whole-body PEI from postoperative week 2 through month 3. This interval was defined based on the assessment schedule and the clinical aim of characterizing subacute recovery after the immediate postoperative phase. PEI was transformed as log (PEI+1). A linear mixed-effects model fitted by restricted maximum likelihood included fixed intercept and linear time effects and patient-specific random intercepts and slopes with an unstructured covariance matrix. Time was the exact number of weeks after surgery and was centered at the week-2 assessment. Slopes were standardized by the cohort SD, effect estimates are reported per 1-SD slower contraction. Model convergence, singularity, scaled gradients, residuals, influence, random-effect variances and correlation, and conditional slope reliability were examined.

### Outcomes and covariates

The primary outcome was moderate-to-severe operated-knee pain at 12 months (12-month pain), requiring an average knee NRS score of 4 or higher, pain on at least 4 days per week, continuous or recurrent pain for at least the preceding 3 months, and pain within the operated knee or an anatomically related periarticular region. At each assessment, patients reported the number of days in a typical week during the preceding 3 months on which pain was present in or around the operated knee (0–7 days) and whether this pain had been present continuously or had recurred repeatedly for at least 3 months (yes/no).

Secondary outcomes were operated-knee NRS at 6 and 12 months; Knee injury and Osteoarthritis Outcome Score for Joint Replacement (KOOS-JR) and Forgotten Joint Score-12 at 12 months; failure to achieve a clinically important KOOS-JR improvement of at least 14 points from baseline; and time from the 3-month landmark to first reoperation or revision, loss to follow-up, or 12 months after surgery.

Baseline variables included age, sex, body mass index (BMI), smoking, education, Charlson Comorbidity Index, diabetes, chronic low back pain, contralateral knee or hip osteoarthritis, fibromyalgia or another chronic widespread pain condition, preoperative opioid use, knee-pain duration, operated-knee NRS, whole-body PEI, KOOS-JR, number of painful regions, painDETECT-defined neuropathic-like pain, Pain Catastrophizing Scale score, and Hospital Anxiety and Depression Scale (HADS) scores. Treatment variables included Kellgren-Lawrence grade, coronal alignment, implant type, patellar resurfacing, surgical approach, tourniquet use, anesthesia, regional nerve block, length of stay, and complications before the landmark.

Postoperative care followed a standardized institutional pathway. Multimodal analgesia included regional nerve block, nonopioid analgesics, and rescue opioids for breakthrough pain. Mobilization and physiotherapist-supervised rehabilitation began during hospitalization and included knee range-of-motion exercises, quadriceps strengthening, gait training, and a home-exercise program after discharge.

For patients meeting the primary outcome, alternative explanations for pain were independently adjudicated by two arthroplasty surgeons blinded to the spatial-contraction measurements, using the clinical history, physical examination, postoperative radiographs, and, when indicated, inflammatory markers, joint aspiration, stress radiographs, or computed tomography; disagreements were resolved by consensus.

### Statistical analysis

Continuous variables were summarized as mean (SD) or median (interquartile range, IQR), and categorical variables as number (percentage). Baseline differences were described using absolute standardized mean differences (SMDs). Postoperative PEI trajectories by outcome status were estimated with a generalized additive mixed-effects model.

The association between the standardized continuous PEI slope and the primary outcome was estimated using sequential logistic regression. Model 1 was unadjusted. The Model 2 adjusted for age, sex, BMI, Charlson Comorbidity Index, chronic low back pain, fibromyalgia or another chronic widespread pain condition, preoperative opioid use, preoperative operated-knee NRS, preoperative whole-body PEI, preoperative KOOS-JR, painDETECT-defined neuropathic-like pain, Pain Catastrophizing Scale score, Hospital Anxiety and Depression Scale anxiety and depression scores, and any complication before the 3-month assessment. Model 3 added week-2 PEI and knee NRS; Model 4 added month-3 PEI and knee NRS to Model 2; and Model 5 added both week-2 and month-3 PEI and NRS. Postoperative PEI was entered as log (PEI+1), and NRS was continuous. Odds ratios (ORs) with 95% confidence intervals (CIs) were reported. Likelihood-ratio tests compared each model with an otherwise identical model omitting the PEI slope. A Firth-penalized version of Model 5 assessed small-sample bias and model stability. Collinearity in Model 5 was assessed using variance inflation factors (VIFs).

A Model 2-adjusted restricted cubic spline with knots at the 5th, 35th, 65th, and 95th percentiles and the median slope as reference characterized the continuous dose-response relation; likelihood-ratio tests evaluated the overall and nonlinear components. Secondary continuous outcomes were analyzed by linear regression and failure of clinically important KOOS-JR improvement by logistic regression.

Sensitivity analyses substituted absolute PEI change, relative contraction rate, time-weighted PEI area under the curve, operated-lower-extremity PEI slope, operated-knee PEI slope, or intensity-weighted pain-area slope for the primary exposure and repeated Model 2. The primary analysis was also repeated after excluding outcome events with an adjudicated alternative explanation. To propagate slope-estimation uncertainty, a Bayesian shared-parameter joint model simultaneously fitted a normal longitudinal submodel for log (PEI+1) and a Bernoulli-logit outcome submodel with Model 2 covariates, linking them through the standardized latent patient-specific slope. Weakly informative normal, half-Student-t, and LKJ priors were used. Four chains of 6,000 iterations (3,000 warm-up) were fitted with cmdstanr 0.8.1 and CmdStan 2.36.0; R-hat, effective sample size, divergent transitions, treedepth, and posterior predictive checks assessed computation and fit.

Missingness was reported for each exposure, covariate, and outcome. Analyzed patients were compared with otherwise eligible nonanalyzed patients using absolute SMDs. Missing covariates were imputed by chained equations in 50 datasets. All conventional and Firth-penalized multivariable logistic models were fitted separately in each imputed dataset. Log-odds coefficients and their variance estimates were combined using Rubin's rules; accordingly, the Firth 95% CI was an MI-pooled Wald-type interval. Two logistic models were fitted in each imputed dataset to estimate the probabilities of exposure and outcome observation. Positivity, weight distributions, effective sample size, and covariate balance were examined; weighted logistic models used robust sandwich standard errors, and estimates were combined using Rubin's rules.

Acquisition-method robustness was assessed by adjusting Model 2 for paper versus electronic acquisition and testing a slope-by-method interaction. Analyses were performed in R 4.4.2. All frequentist tests were two-sided, with *P* < 0.05 considered statistically significant.

## Results

### Study population

Of 812 patients screened, 72 did not meet the eligibility criteria, 10 did not reach the postoperative 3-month landmark, 71 had insufficient early postoperative pain drawings, 35 had no ascertainable 12-month pain outcome. The final 3-month landmark cohort comprised 624 patients (Supplementary Figure S2), of whom 92 (14.7%) had 12-month pain (Supplementary Table S1).

The 106 otherwise eligible patients excluded because of incomplete exposure or outcome observation were slightly older, had higher preoperative pain and psychological-distress measures, and were more likely to have lower educational attainment, greater comorbidity, and paper-based pain-map acquisition than analyzed patients. The largest absolute SMD was 0.23 for paper-based acquisition; all other absolute SMDs were 0.22 or lower. Covariate missingness in the analyzed cohort ranged from 0% to 1.9%; missing covariates were multiply imputed (Supplementary Table S2).

### Pain-drawing processing and reproducibility

Among the 1,872 early postoperative pain drawings, 39 (2.1%) required secondary quality-control review because of residual annotations or other nonpain markings (*n* = 18), landmark-placement or registration errors (*n* = 12), or scanning, background, or cropping artifacts (*n* = 9) (Supplementary Table S3).

Reproducibility was assessed in 375 drawings from 125 patients, including 234 paper-based drawings from 78 patients and 141 electronic drawings from 47 patients. The inter-rater absolute-agreement ICC for PEI was 0.987 (95% CI, 0.983–0.990), the median Dice coefficient was 0.949 (IQR, 0.927–0.968), and the mean absolute PEI difference was 0.24 percentage points; the Bland–Altman mean difference was 0.03 percentage points, with 95% limits of agreement from −0.59 to 0.65. Corresponding intra-rater estimates were an ICC of 0.994 (95% CI, 0.992–0.996), a median Dice coefficient of 0.971 (IQR, 0.956–0.983), a mean absolute difference of 0.14 percentage points, and a mean difference of 0.01 percentage points (95% limits of agreement, −0.37–0.39). Inter- and intra-rater ICCs for the early PEI slope were 0.982 (95% CI, 0.974–0.988) and 0.991 (95% CI, 0.987–0.994), respectively (Supplementary Table S3).

### Pain extent index distributions and early spatial contraction

Median whole-body PEI increased from 4.1% (IQR, 2.2%–7.0%) preoperatively to 6.8% (IQR, 4.2%–10.3%) at 2 weeks, then decreased to 4.7% (IQR, 2.6%–7.8%) at 6 weeks, 2.8% (IQR, 1.1%–5.3%) at 3 months, 2.0% (IQR, 0.5%–4.2%) at 6 months, and 1.6% (IQR, 0.2%–3.8%) at 12 months. Patients who developed 12-month pain had greater preoperative PEI than those who did not (median, 6.7% vs 3.8%) and greater residual pain extent at month 3 (6.1% vs 2.3%) (Table 1).

**Table 1:** Whole-body PEI distributions over time.

Assessment*	Overall	No 12-month pain	12-month pain
Preoperative	4.1 (2.2–7.0)	3.8 (2.1–6.5)	6.7 (3.8–10.4)
Postoperative week 2	6.8 (4.2–10.3)	6.5 (4.0–9.8)	8.9 (5.9–13.1)
Postoperative week 6	4.7 (2.6–7.8)	4.3 (2.4–7.0)	7.4 (4.8–10.9)
Postoperative month 3	2.8 (1.1–5.3)	2.3 (0.8–4.4)	6.1 (3.9–9.3)
Postoperative month 6	2.0 (0.5–4.2)	1.5 (0.2–3.0)	5.8 (3.6–8.7)
Postoperative month 12	1.6 (0.2–3.8)	1.1 (0.0–2.4)	5.6 (3.5–8.4)
Early contraction velocity, units/week	−0.067 (0.039)	−0.073 (0.035)	−0.033 (0.036)
Relative contraction rate, %	56.8 (37.4–72.1)	61.7 (45.6–75.0)	28.4 (10.2–45.8)
Spatial pain expansion	44 (7.1)	25 (4.7)	19 (20.7)

PEI values and relative contraction rates are median (IQR); velocity estimates are mean (SD); spatial pain expansion is number (%). More negative velocity values indicate faster spatial contraction. Spatial pain expansion was defined as a patient-specific slope greater than zero.

* The No 12-month Pain and 12-month Pain cohorts initially consisted of 532 and 92 patients, respectively, through the 3-month follow-up. The sample sizes were 509 and 88 at the 6-month assessment and 501 and 88 at the 12-month assessment, respectively.

Overall-cohort heat maps showed pain concentrated around the operated knee preoperatively, with less frequent involvement of the ipsilateral lower extremity, contralateral knee, lower back, and other extra-articular regions. At 2 weeks, pain density increased around the operated knee and extended into the adjacent thigh and lower leg; it contracted substantially by 3 months and declined more gradually thereafter, with residual pain at 6 and 12 months concentrated mainly around the operated knee (Figure 1A). The generalized additive mixed-effects model showed a nonlinear postoperative trajectory that differed by 12-month pain status (time-by-pain-status smooth interaction, *P* < 0.001). PEI declined steeply between 2 weeks and 3 months and then approached a low plateau among patients without 12-month pain, whereas those with 12-month pain had a shallower early decline and persistently higher PEI from 3 to 12 months (Figure 1B).

**Figure 1:**
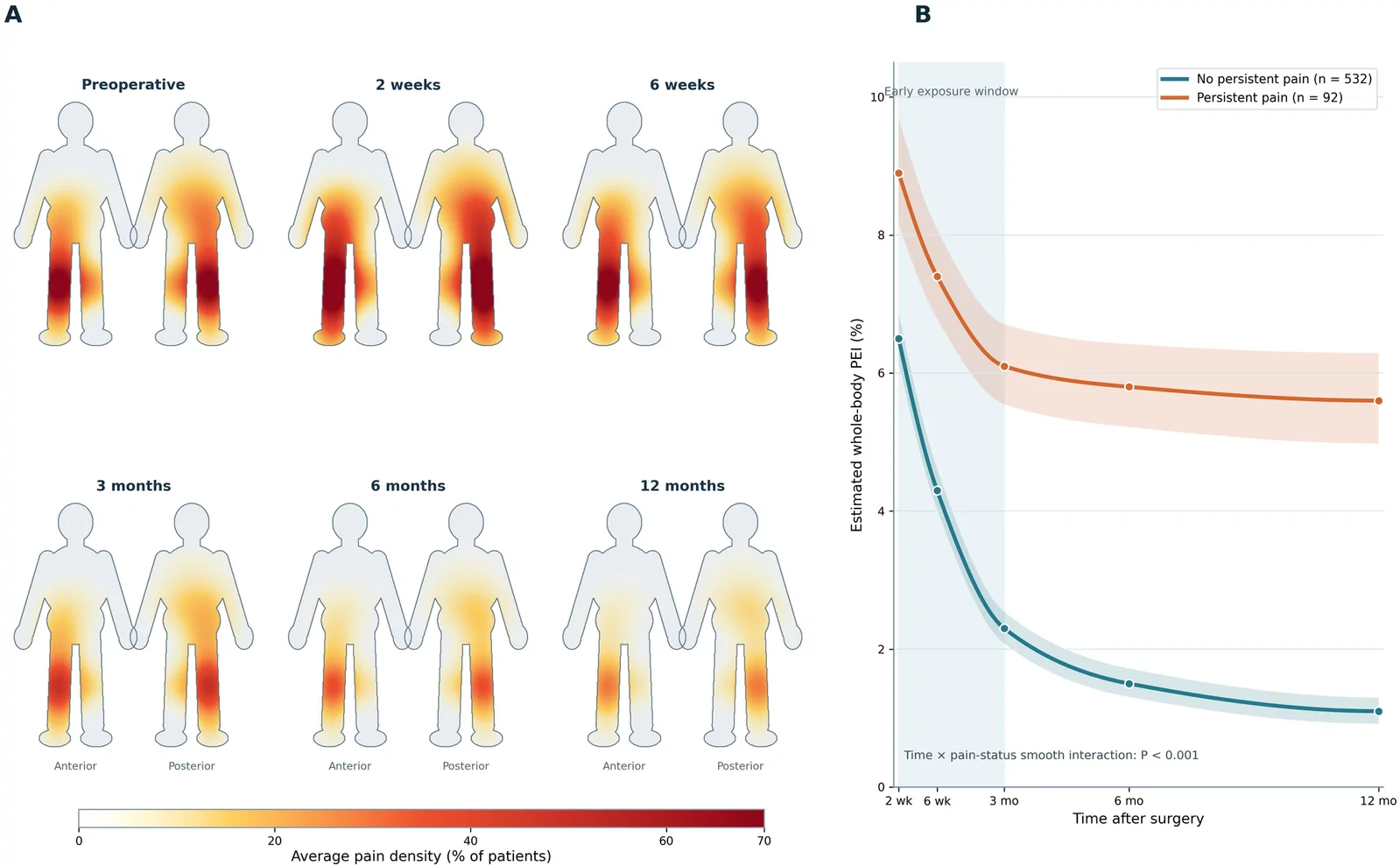
Cohort-level pain-density maps and pain-status–specific PEI trajectories. **(A)** Overall-cohort average pain-density heat maps at each assessment, pooling patients with and without moderate-to-severe persistent knee pain at 12 months. Increasing color intensity indicates a greater proportion of the overall cohort reporting pain at the corresponding anatomical location. **(B)** Generalized additive mixed-effects model estimates of postoperative whole-body PEI according to moderate-to-severe persistent knee pain status at 12 months. Lines represent estimated trajectories, shaded bands represent 95% confidence intervals, and points indicate assessment times. PEI, Pain Extent Index.

The population-average fixed-effect slope between week 2 and month 3 was −0.067 log (PEI+1) units/week (95% CI, −0.070 to −0.064; *P* < 0.001). Patient-specific slopes had a median of −0.065 units/week (IQR, −0.090 to −0.043) and an SD of 0.039. The mixed-effects model included 1,872 observations from 624 patients; the random-intercept, random-slope, and residual variances were 0.276 (SD, 0.525), 0.0021 (SD, 0.046 units/week), and 0.041 (SD, 0.202), respectively, and the intercept–slope correlation was −0.42. The SD of the empirical Bayes slopes was 0.039 versus a latent random-slope SD of 0.046, with a median conditional slope reliability of 0.75 (IQR, 0.70–0.79) (Supplementary Table S4).

Mean contraction velocity was −0.073 units/week (SD, 0.035) among patients without 12-month pain and −0.033 units/week (SD, 0.036) among those with 12-month pain. Positive slopes occurred in 44 patients (7.1%): 25 of 532 (4.7%) without and 19 of 92 (20.7%) with 12-month pain. The median relative Spatial Pain Contraction Rate was 56.8% (IQR, 37.4%–72.1%) overall, 61.7% (IQR, 45.6%–75.0%) among patients without 12-month pain, and 28.4% (IQR, 10.2%–45.8%) among those with 12-month pain (Table 1).

### Primary outcome analysis

Each 1-SD slower spatial contraction was associated with higher odds of 12-month pain in the unadjusted model (OR, 2.46; 95% CI, 1.97–3.07) and after adjustment for baseline and perioperative covariates (adjusted OR, 1.96; 95% CI, 1.51–2.54). The association persisted after additional adjustment for week-2 PEI and knee NRS (adjusted OR, 1.72; 95% CI, 1.29–2.29), month-3 PEI and knee NRS (adjusted OR, 1.48; 95% CI, 1.10–1.99), and static PEI and knee NRS at both time points (adjusted OR, 1.42; 95% CI, 1.03–1.95; *P* = 0.031; likelihood-ratio *P* = 0.035; Table 2).

**Table 2:** Association between early spatial pain-contraction velocity and moderate-to-severe knee pain 12 months after total knee arthroplasty after sequential adjustment for static pain burden.

Model	Adjustment	OR per 1-SD slower contraction (95% CI)	P value	LR *P* value*
Model 1	Unadjusted	2.46 (1.97–3.07)	<0.001	<0.001
Model 2	Baseline and perioperative covariates	1.96 (1.51–2.54)	<0.001	<0.001
Model 3	Model 2 plus week-2 PEI and knee NRS	1.72 (1.29–2.29)	<0.001	<0.001
Model 4	Model 2 plus month-3 PEI and knee NRS	1.48 (1.10–1.99)	0.01	0.013
Model 5	Model 2 plus week-2 and month-3 PEI and knee NRS	1.42 (1.03–1.95)	0.031	0.035

Values are ORs per 1-SD increase in the patient-specific PEI slope; higher slopes indicate slower spatial pain contraction. Model 2 adjusted for age, sex, body mass index, Charlson Comorbidity Index, chronic low back pain, fibromyalgia or another chronic widespread pain condition, preoperative opioid use, preoperative operated-knee NRS, preoperative whole-body PEI, preoperative KOOS-JR, painDETECT-defined neuropathic-like pain, Pain Catastrophizing Scale score, Hospital Anxiety and Depression Scale anxiety and depression scores, and any complication before the 3-month assessment. Postoperative PEI was entered as log (PEI+1).

* Likelihood-ratio test comparing otherwise identical models with versus without the continuous PEI slope.

BMI, body mass index; CI, confidence interval; KOOS-JR, knee injury and osteoarthritis outcome score for joint replacement; LR, likelihood ratio; NRS, numerical rating scale; OR, odds ratio; PEI, pain extent index.

The corresponding MI-pooled Firth-penalized estimate for Model 5 was similar (OR, 1.39; 95% CI, 1.02–1.90; *P* = 0.038). The VIF for the spatial-contraction slope ranged from 174.8 to 181.6 across the 50 imputed datasets; the maximum VIF for any Model 5 predictor was 191.3. Restricted cubic spline analysis showed an overall association between the continuous PEI slope and 12-month knee pain (*P* < 0.001), with evidence of nonlinearity (*P* = 0.019); adjusted odds increased progressively as the slope approached zero (Figure 2).

**Figure 2:**
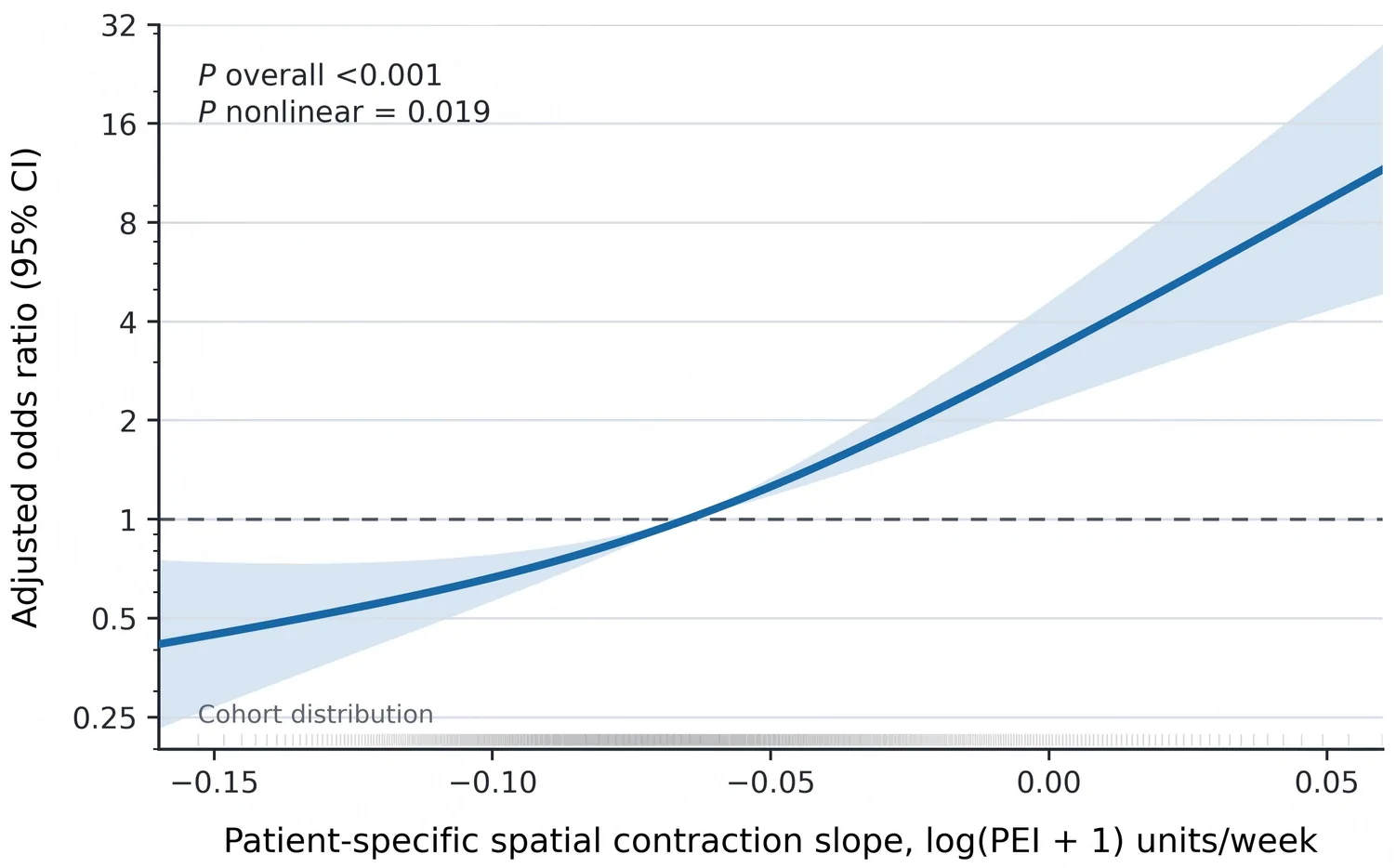
Continuous dose–response association between early spatial pain-contraction velocity and postsurgical knee pain. The curve shows the adjusted restricted cubic spline association between the patient-specific spatial contraction slope and moderate-to-severe knee pain at 12 months. The solid line represents the adjusted odds ratio and the shaded area the 95% confidence interval. The horizontal dashed line indicates an odds ratio of 1.0, and the rug shows the distribution of individual slopes. The cohort median of −0.065 log(PEI+1) units/week was used as the reference. The overall association was significant (*P* < 0.001), with evidence of nonlinearity (*P* = 0.019). OR, odds ratio; PEI, pain extent index.

### Secondary outcomes

Each 1-SD slower contraction was associated with higher operated-knee NRS scores at 6 months (adjusted *β*, 0.39; 95% CI, 0.27–0.51) and 12 months (adjusted *β*, 0.43; 95% CI, 0.30–0.56), and with lower 12-month KOOS-JR (adjusted *β*, −4.58; 95% CI, −5.78 to −3.38) and Forgotten Joint Score-12 scores (adjusted *β*, −6.31; 95% CI, −8.14 to −4.48; all *P* < 0.001). Slower contraction was also associated with higher odds of failing to achieve clinically important KOOS-JR improvement (adjusted OR, 1.46; 95% CI, 1.18–1.80; *P* < 0.001). Nine patients underwent reoperation or revision; the exploratory univariable time-to-event analysis was imprecise (HR per 1-SD slower contraction, 1.70; 95% CI, 0.95–3.03; *P* = 0.073; Table 3).

**Table 3:** Association of early spatial pain-contraction velocity with secondary clinical outcomes during the 12-month follow-up.

Outcome	Overall value	Effect measure	Unadjusted effect per 1-SD slower contraction (95% CI)	Adjusted effect per 1-SD slower contraction (95% CI)	Adjusted *P* value
Operated-knee NRS at 6 months (*n* = 610)	2.1 (1.6)	β	0.56 (0.44 to 0.68)	0.39 (0.27 to 0.51)	<0.001
Operated-knee NRS at 12 months (*n* = 624)	1.8 (1.7)	β	0.61 (0.48 to 0.74)	0.43 (0.30 to 0.56)	<0.001
KOOS-JR at 12 months (*n* = 607)	81.9 (14.2)	β	−6.70 (−7.78 to −5.62)	−4.58 (−5.78 to −3.38)	<0.001
Forgotten Joint Score-12 at 12 months (*n* = 602)	73.9 (20.4)	β	−9.42 (−10.98 to −7.86)	−6.31 (−8.14 to −4.48)	<0.001
Failure to achieve clinically important KOOS-JR improvement (*n* = 606)	127 (21.0%)	OR	1.79 (1.47 to 2.18)	1.46 (1.18 to 1.80)	<0.001
Time to first reoperation or revision within 12 months* (*n* = 624)	9 events (1.4%)	HR	1.70 (0.95 to 3.03)	—†	0.073

Values are mean (SD) or number (%). Effects are reported per 1-SD increase in the patient-specific PEI slope.

Adjusted models included age, sex, body mass index, Charlson Comorbidity Index, chronic low back pain, fibromyalgia or another chronic widespread pain condition, preoperative opioid use, preoperative operated-knee NRS, preoperative whole-body PEI, preoperative KOOS-JR, painDETECT-defined neuropathic-like pain, Pain Catastrophizing Scale score, Hospital Anxiety and Depression Scale anxiety and depression scores, and any complication before the 3-month assessment.

* The median time from the 3-month landmark to reoperation or revision was 4.6 months (IQR, 2.2–7.1 months).

† Because only nine events occurred, an exploratory univariable Cox model was used to avoid overfitting; therefore, no multivariable-adjusted HR is reported.

β, regression coefficient; BMI, body mass index; CI, confidence interval; HR, hazard ratio; KOOS-JR, Knee injury and osteoarthritis outcome score for joint replacement; NRS, numerical rating scale; OR, odds ratio; PEI, pain extent index.

### Sensitivity and robustness analyses

Results were consistent when the exposure was defined as absolute PEI change (adjusted OR per 1-SD less-favorable change, 1.82; 95% CI, 1.45–2.29), relative Spatial Pain Contraction Rate (adjusted OR per 1-SD lower rate, 1.74; 95% CI, 1.38–2.19), or time-weighted PEI area under the curve (adjusted OR per 1-SD higher area, 1.89; 95% CI, 1.49–2.39). Comparable associations were observed for the operated lower-extremity PEI slope (adjusted OR, 1.71; 95% CI, 1.36–2.15), operated-knee PEI slope (adjusted OR, 1.65; 95% CI, 1.31–2.08), and intensity-weighted pain-area slope (adjusted OR, 1.84; 95% CI, 1.45–2.34). In the shared-parameter joint model, each 1-SD slower latent spatial contraction was associated with a posterior OR of 2.05 (95% credible interval, 1.54–2.73); all R-hat values were <1.01 (Supplementary Table S4).

Twelve of the 92 patients meeting the primary outcome had an adjudicated mechanical, prosthesis-related, infectious, or other alternative explanation for pain. After their exclusion, slower spatial contraction remained associated with 12-month pain (adjusted OR per 1 SD, 1.89; 95% CI, 1.42–2.52; *P* < 0.001; Supplementary Table S4).

Among the 730 clinically eligible landmark patients, 659 (90.3%) had an estimable early PEI slope, of whom 624 (94.7%) had an ascertainable 12-month outcome. Estimated probabilities ranged from 0.51 to 0.98 for exposure observation and from 0.70 to 1.00 for outcome observation. Before truncation, stabilized weights had a mean of 1.003 (SD, 0.22), a range of 0.88–2.08, and 1st and 99th percentiles of 0.89 and 1.74. After truncation, the mean was 0.998 (SD, 0.18), the range was 0.89–1.74, and the effective sample size was 604; the maximum absolute SMD decreased from 0.23 before weighting to 0.06 after weighting. The weighted association (OR per 1-SD slower contraction, 1.91; 95% CI, 1.46–2.50; *P* < 0.001) was similar to the primary unweighted estimate (Supplementary Table S2).

Paper-based drawings were used for 387 patients, including 56 outcome events, and electronic drawings for 237 patients, including 36 events. After adjustment for acquisition method, the association remained evident overall (adjusted OR per 1-SD slower contraction, 1.95; 95% CI, 1.50–2.53; *P* < 0.001) and was similar for paper-based (adjusted OR, 1.91; 95% CI, 1.39–2.63) and electronic drawings (adjusted OR, 2.04; 95% CI, 1.34–3.12; P for interaction=0.79) (Supplementary Table S3).

## Discussion

The most important finding was that patients whose spatial pain burden contracted more slowly between 2 weeks and 3 months after TKA were more likely to have moderate-to-severe knee pain at 12 months. This association persisted after accounting for baseline risk and for pain extent and knee pain intensity at both ends of the recovery window, indicating that rate of change carried information beyond static snapshots. Slower contraction also accompanied worse later pain and joint-related outcomes. Thus, early spatial contraction appears to be a reproducible candidate recovery marker, although the study does not establish causality or a stand-alone prediction tool.

Long-term TKA studies show that unfavorable pain and function courses can remain separated for years (2). Perioperative insomnia trajectories also relate to later function, underscoring that recovery is multidimensional and changes over time (7). Our study is concordant with this framework but quantifies change in anatomical pain extent during subacute recovery. The residual association after adjustment for week-2 and month-3 PEI and NRS suggests that a slope captures information not contained in either visit alone. The exposure window also bridges acute and established chronic pain. Research on subacute pain after TKA has focused largely on the first postoperative week and preoperative predictors of high-pain response (8). Daily remote monitoring shows that dense symptom collection is feasible, but it has mainly assessed conventional recovery measures or treatment comparisons (9). Immediate pain is strongly influenced by tissue injury and analgesia; the week-2 to month-3 interval may better reflect resolution of inflammation, restoration of movement, and normalization of pain processing. This timing may explain why spatial contraction added information beyond early intensity measures.

Our findings complement, rather than replace, established baseline predictors. Preoperative pain distribution and self-reported central sensitization have predicted worse 1-year pain, while hyperalgesia and neuropathic qualities have shown variable prognostic value (3, 4). Catastrophizing, anxiety, and depression are also associated with poor TKA outcomes (5, 10). High pain despite less severe radiographic disease may indicate discordance between joint pathology and symptoms, whereas multivariable risk indices show incomplete transportability (11, 12). Slower postoperative contraction remained associated with later pain after these domains were considered, suggesting that serial spatial assessment may update presurgical risk by measuring actual recovery. The spatial findings are plausible but not mechanism-specific. Knee osteoarthritis phenotyping identifies groups defined by different combinations of local symptoms, widespread pain, and sensitization (13). The IASP nociplastic framework likewise emphasizes that mechanism cannot be inferred from intensity alone (14). Central sensitization may connect psychological distress with poor arthroplasty outcomes, while exploratory imaging and sensory studies implicate synovitis, altered nociceptive processing, and catastrophizing (15, 16). Inflammatory biomarker work further suggests biological heterogeneity (17). Slower PEI reduction could therefore reflect persistent peripheral input, guarding, referred pain, or sustained nervous-system amplification. The present data cannot distinguish these pathways.

Anatomical information may also explain the associations with several later outcomes. Persistent post-TKA pain varies in quality and distribution, and detailed descriptors reveal patterns missed by intensity scores (18). Neuropathic features may be diffuse, radiating, or accompanied by sensory abnormalities (19). A whole-body slope is unlikely to represent one phenotype, but may integrate improvement across the knee, adjacent limb, and remote sites. Expectations can shape pain and function over time, so behavioral engagement may influence both drawing patterns and recovery (20). These explanations remain hypotheses because pain drawings show where pain is experienced, not its biological cause.

The principal novelty is the separation of dynamic spatial recovery from static burden. Patients with the same PEI at one visit may have reached it through rapid contraction, persistent pain, or spatial expansion. Estimating a patient-specific slope and retaining an association after adjustment for both endpoint PEI and NRS values operationalizes that distinction. Consistency across whole-body, operated-limb, operated-knee, and intensity-weighted measures indicates that the signal was not confined to one spatial definition. The nonlinear association suggests that failure to contract may be most informative, but uncertainty at the extremes precludes a treatment threshold.

Clinically, serial pain drawings could complement NRS and joint-specific questionnaires at routine follow-up. Limited contraction could prompt structured reassessment of the wound and prosthesis, infection or mechanical problems, range of motion, rehabilitation, contralateral disease, low-back pain, and neuropathic features. Digital collection could fit remote monitoring pathways that already support repeated recovery assessment (9). The result should not trigger automatic imaging, treatment, or revision. Revision outcomes differ by the underlying diagnosis, and unexplained pain requires careful evaluation before further surgery (21). Spatial contraction is a signal to look more closely, not a diagnosis.

The signal may also focus nonoperative care. Radiating pain could prompt neuropathic assessment, whereas widespread residual pain may justify review of sleep, mood, catastrophizing, analgesic use, and rehabilitation barriers. Neuropathic features remain common in chronic post-TKA pain, and persistent opioid use has multiple preoperative and postoperative determinants (19, 22). Any protocol should combine the drawing with examination and validated patient-reported measures. Processing was reproducible and performed similarly for paper and electronic acquisition in this cohort, supporting pragmatic use; implementation studies must still assess burden, acceptability, and whether feedback changes care.

Identifying an adverse trajectory does not identify the correct intervention. In established chronic post-TKA pain, adding neuromuscular exercise to pain neuroscience education was not superior to education alone (23). Secondary analyses also emphasize heterogeneity in pain mechanisms and psychosocial characteristics (24). These findings argue for mechanism-informed triage and targeted treatment trials, rather than a uniform escalation of rehabilitation for every patient with slow contraction.

Several limitations warrant caution. This single-center cohort may not represent other demographic, cultural, or care-pathway settings. The retrospective analysis of prospectively collected data was observational, so residual confounding and unmeasured treatment changes remain possible. A linear slope from three assessments summarized a potentially nonlinear process, and empirical Bayes shrinkage may have attenuated between-patient differences. Pain drawings depend on comprehension, recall, and drawing behavior; image registration reduces processing variation but cannot assign a pain mechanism. Weighting supported robustness but depends on correctly specified observation models and cannot address unmeasured selection. The primary outcome was symptom-defined, although excluding documented alternative explanations did not materially change the result. Reoperation analyses were underpowered, follow-up ended at 12 months, and no external validation, calibration, incremental discrimination, or actionable cutoff was evaluated.

Future work should prospectively validate the slope in multicenter cohorts with broader demographic and health-system representation. Standardized digital drawings and remote monitoring could permit denser sampling and comparison of nonlinear or pattern-based trajectories with the simpler three-visit slope (9). Studies should test incremental value over NRS, KOOS-JR, psychosocial measures, and routine clinical findings using prespecified calibration and decision-curve analyses. Mechanistic cohorts should pair spatial trajectories with sensory testing, inflammatory markers, sleep, activity, and neuropathic phenotyping. A stratified trial should then test whether an early spatial-contraction alert linked to predefined diagnostic and treatment pathways improves outcomes, while allowing for the intervention heterogeneity suggested by current trial evidence (23, 24).

In conclusion, slower contraction of spatial pain extent during subacute recovery was consistently associated with moderate-to-severe knee pain and poorer patient-reported outcomes after TKA, after adjustment for static pain burden. The findings support a dynamic view in which the direction and speed of recovery matter, but they do not establish causality or justify stand-alone decisions. If externally validated and shown to add useful predictive value, serial pain drawings may offer a practical way to identify patients who need earlier, more comprehensive assessment.

## Data Availability

The original contributions presented in the study are included in the article/Supplementary Material, further inquiries can be directed to the corresponding author.

## Supplementary material

SUPPLEMENTARY FIGURE S1 Standardized whole-body pain drawing. Patients shaded all areas in which they had experienced pain during the preceding 7 days on anterior and posterior body silhouettes. Right and left refer to the patient's anatomical orientation.

SUPPLEMENTARY FIGURE S2 Formation of the 3-month landmark cohort. PEI, Pain Extent Index. Exclusion categories were hierarchical and mutually exclusive.
