# Supplementary material for: Early spatial pain contraction and moderate-to-severe knee pain 12 months after total knee arthroplasty: a retrospective cohort study
Source: Front Pain Res (Lausanne). 2026 Oct 12;7:1948322. doi: 10.3389/fpain.2026.1948322 (PMC13696397; doi:10.3389/fpain.2026.1948322)
Supplement: Supplementary file 1 [file Table1.docx]

Supplementary Table 1. Characteristics of the study population according to 12-month pain status.

| **Characteristic** | **Overall (n=624)** | **No 12-month pain (n=532)** | **12-month pain (n=92)** | **Absolute SMD** |
| --- | --- | --- | --- | --- |
| Age, years | 66.9 (7.0) | 67.1 (6.8) | 65.7 (7.5) | 0.196† |
| Female sex | 401 (64.3) | 333 (62.6) | 68 (73.9) | 0.245† |
| BMI, kg/m² | 27.3 (3.9) | 27.1 (3.8) | 28.4 (4.2) | 0.325† |
| Current smoking | 55 (8.8) | 46 (8.6) | 9 (9.8) | 0.039 |
| Middle-school education or lower | 332 (53.2) | 275 (51.7) | 57 (62.0) | 0.208† |
| Charlson Comorbidity Index ≥2 | 138 (22.1) | 112 (21.1) | 26 (28.3) | 0.168† |
| Diabetes mellitus | 119 (19.1) | 97 (18.2) | 22 (23.9) | 0.140† |
| Chronic low back pain | 152 (24.4) | 119 (22.4) | 33 (35.9) | 0.301† |
| Contralateral knee or hip osteoarthritis | 200 (32.1) | 165 (31.0) | 35 (38.0) | 0.148† |
| Fibromyalgia or another chronic widespread pain condition | 27 (4.3) | 17 (3.2) | 10 (10.9) | 0.304† |
| Preoperative opioid use | 51 (8.2) | 36 (6.8) | 15 (16.3) | 0.302† |
| Operated-knee NRS score | 6.6 (1.4) | 6.5 (1.4) | 7.1 (1.3) | 0.444† |
| Whole-body PEI, % | 4.1 (2.2–7.0) | 3.8 (2.1–6.5) | 6.7 (3.8–10.4) | 0.520† |
| Duration of operated-knee pain, years | 5.0 (3.0–8.0) | 5.0 (3.0–8.0) | 7.0 (4.0–10.0) | 0.340† |
| KOOS-JR score | 49.5 (13.1) | 50.3 (12.8) | 44.7 (13.4) | 0.427† |
| Number of painful anatomical regions | 3 (2–5) | 3 (2–5) | 5 (3–7) | 0.490† |
| Neuropathic-like pain, painDETECT ≥13 | 106 (17.0) | 77 (14.5) | 29 (31.5) | 0.414† |
| Pain Catastrophizing Scale score | 16.3 (10.1) | 15.6 (9.8) | 20.6 (10.7) | 0.487† |
| HADS anxiety score | 6.3 (3.6) | 6.0 (3.4) | 8.2 (4.0) | 0.593† |
| HADS depression score | 6.0 (3.6) | 5.7 (3.5) | 7.4 (3.9) | 0.459† |
| Kellgren–Lawrence grade |  |  |  | 0.089 |
| Grade II | 20 (3.2) | 18 (3.4) | 2 (2.2) |  |
| Grade III | 178 (28.5) | 154 (28.9) | 24 (26.1) |  |
| Grade IV | 426 (68.3) | 360 (67.7) | 66 (71.7) |  |
| Preoperative varus alignment, degrees | 6.9 (4.8) | 6.9 (4.7) | 7.1 (5.1) | 0.041 |
| Posterior-stabilized implant | 378 (60.6) | 320 (60.2) | 58 (63.0) | 0.06 |
| Patellar resurfacing | 232 (37.2) | 196 (36.8) | 36 (39.1) | 0.047 |
| Medial parapatellar approach | 602 (96.5) | 513 (96.4) | 89 (96.7) | 0.017 |
| Tourniquet use | 533 (85.4) | 453 (85.2) | 80 (87.0) | 0.052 |
| Spinal anesthesia | 503 (80.6) | 430 (80.8) | 73 (79.3) | 0.037 |
| Regional nerve block | 494 (79.2) | 420 (78.9) | 74 (80.4) | 0.037 |
| Standardized multimodal analgesia pathway prescribed‡ | 624 (100) | 532 (100) | 92 (100) | NA |
| Standardized rehabilitation pathway prescribed‡ | 624 (100) | 532 (100) | 92 (100) | NA |
| Hospital length of stay, days | 6 (5–8) | 6 (5–8) | 6 (5–8) | 0.06 |
| Complication before 3-month assessment | 37 (5.9) | 29 (5.5) | 8 (8.7) | 0.127† |
| Paper-based pain-map acquisition | 387 (62.0) | 331 (62.2) | 56 (60.9) | 0.028 |

Values are mean (SD), median (IQR), or number (%). SMDs are presented as absolute values.

†Absolute SMD >0.10, indicating a potentially important between-group difference.

‡All participants were prescribed the institutional postoperative care pathways. Multimodal analgesia included regional nerve block when indicated, nonopioid analgesics unless contraindicated, and rescue opioids for breakthrough pain. Rehabilitation included mobilization during hospitalization, knee range-of-motion exercises, quadriceps strengthening, gait training, and a home-exercise program after discharge. SMDs were not calculated because there was no between-patient variation in pathway prescription.

Abbreviations: BMI, body mass index; HADS, Hospital Anxiety and Depression Scale; IQR, interquartile range; KOOS-JR, Knee injury and Osteoarthritis Outcome Score for Joint Replacement; NA, not applicable; NRS, numerical rating scale; PEI, Pain Extent Index; SMD, standardized mean difference.
