# Supplementary material for: Early spatial pain contraction and moderate-to-severe knee pain 12 months after total knee arthroplasty: a retrospective cohort study
Source: Front Pain Res (Lausanne). 2026 Oct 12;7:1948322. doi: 10.3389/fpain.2026.1948322 (PMC13696397; doi:10.3389/fpain.2026.1948322)
Supplement: Supplementary file 2 [file Table2.docx]

Supplementary Table 2. Cohort selection, missingness, and inverse-probability-of-observation weighting diagnostics.

| **Panel A. Formation of the 3-month landmark cohort** |  |  |
| --- | --- | --- |
| **Cohort-flow category** | **n** | **%** |
| Patients screened | 812 | 100 |
| Did not meet clinical eligibility criteria | 72 | 8.9 |
| Alternative arthroplasty, revision, or simultaneous bilateral procedure | 21 | 2.6 |
| Inflammatory, post-traumatic, or neuropathic arthropathy | 24 | 3 |
| Previous ipsilateral infection or active malignant disease | 9 | 1.1 |
| Severe cognitive impairment | 6 | 0.7 |
| Age younger than 40 years | 3 | 0.4 |
| Contralateral arthroplasty before the landmark | 9 | 1.1 |
| Potentially eligible after clinical exclusions | 740 | 91.1 |
| Did not reach the 3-month landmark | 10 | 1.2 |
| Index-knee reoperation before the landmark | 8 | 1 |
| Death or severe illness before the landmark | 2 | 0.2 |
| Clinically eligible patients reaching the landmark | 730 | 89.9 |
| Insufficient early postoperative pain drawings | 71 | 9.7¹ |
| Week-2 drawing only missing | 14 | 1.9¹ |
| Week-6 drawing only missing | 29 | 4.0¹ |
| Month-3 drawing only missing | 19 | 2.6¹ |
| Two or more early drawings missing | 9 | 1.2¹ |
| Early PEI slope estimable | 659 | 90.3¹ |
| Twelve-month pain outcome unavailable | 35 | 5.3² |
| Unable to contact | 24 | 3.6² |
| Declined 12-month assessment | 7 | 1.1² |
| Severe intercurrent illness | 3 | 0.5² |
| Death after the landmark | 1 | 0.2² |
| Final analysis cohort | 624 | 76.8³ |
| Moderate-to-severe knee pain at 12 Months | 92 | 14.7⁴ |
| No Moderate-to-severe knee pain at 12 Months | 532 | 85.3⁴ |

¹Percentage of the 730 landmark-eligible patients.

²Percentage of the 659 patients with an estimable exposure.

³Percentage of the 812 screened patients.

⁴Percentage of the 624 analyzed patients.

| **Panel B. Comparison of analyzed and otherwise eligible nonanalyzed patients** |  |  |  |
| --- | --- | --- | --- |
| **Characteristic** | **Analyzed cohort (n=624)** | **Observation-excluded cohort (n=106)** | **Absolute SMD** |
| Age, years | 66.9 (7.0) | 68.0 (7.5) | 0.15 |
| Female sex | 401/624 (64.3) | 72/106 (67.9) | 0.08 |
| BMI, kg/m² | 27.3 (3.9) | 27.9 (4.1) | 0.15 |
| Current smoking | 55/621 (8.9) | 12/105 (11.4) | 0.09 |
| Middle-school education or lower | 332/618 (53.7) | 67/104 (64.4) | 0.22 |
| Charlson Comorbidity Index ≥2 | 138/624 (22.1) | 31/106 (29.2) | 0.16 |
| Diabetes mellitus | 119/624 (19.1) | 25/106 (23.6) | 0.11 |
| Chronic low back pain | 152/622 (24.4) | 33/105 (31.4) | 0.16 |
| Fibromyalgia or chronic widespread pain | 27/624 (4.3) | 7/106 (6.6) | 0.1 |
| Preoperative opioid use | 51/623 (8.2) | 13/105 (12.4) | 0.14 |
| Preoperative operated-knee NRS | 6.6 (1.4) | 6.9 (1.5) | 0.21 |
| Preoperative whole-body PEI, % | 4.1 (2.2–7.0) | 4.8 (2.6–8.1) | 0.18 |
| Preoperative KOOS-JR | 49.5 (13.1) | 46.7 (13.8) | 0.21 |
| Neuropathic-like pain, painDETECT ≥13 | 106/614 (17.3) | 25/101 (24.8) | 0.19 |
| Pain Catastrophizing Scale | 16.3 (10.1) | 18.5 (10.7) | 0.21 |
| HADS anxiety score | 6.3 (3.6) | 7.1 (3.9) | 0.21 |
| HADS depression score | 6.0 (3.6) | 6.8 (4.0) | 0.21 |
| Complication before month 3 | 37/624 (5.9) | 10/106 (9.4) | 0.13 |
| Paper-based pain-map acquisition | 387/624 (62.0) | 77/106 (72.6) | 0.23 |

Values are mean (SD), median (IQR), or number/available number (%). SMDs were calculated using available observations.

| **Panel C. Variable-level missingness** | | | |
| --- | --- | --- | --- |
| **Variable** | **Missing in landmark cohort, n/N (%)** | **Missing in analyzed cohort, n/624 (%)** | **Handling** |
| Age | 0/730 (0.0) | 0 (0.0) | Complete |
| Sex | 0/730 (0.0) | 0 (0.0) | Complete |
| BMI | 2/730 (0.3) | 1 (0.2) | Multiple imputation |
| Smoking status | 4/730 (0.5) | 3 (0.5) | Multiple imputation |
| Educational level | 8/730 (1.1) | 6 (1.0) | Multiple imputation |
| Charlson Comorbidity Index | 0/730 (0.0) | 0 (0.0) | Complete |
| Diabetes mellitus | 0/730 (0.0) | 0 (0.0) | Complete |
| Chronic low back pain | 3/730 (0.4) | 2 (0.3) | Multiple imputation |
| Contralateral knee or hip osteoarthritis | 2/730 (0.3) | 1 (0.2) | Multiple imputation |
| Fibromyalgia or another chronic widespread pain condition | 1/730 (0.1) | 0 (0.0) | Multiple imputation |
| Preoperative opioid use | 2/730 (0.3) | 1 (0.2) | Multiple imputation |
| Preoperative knee NRS | 4/730 (0.5) | 2 (0.3) | Multiple imputation |
| Preoperative whole-body PEI | 0/730 (0.0) | 0 (0.0) | Complete |
| Duration of knee pain | 12/730 (1.6) | 8 (1.3) | Multiple imputation |
| Preoperative KOOS-JR | 7/730 (1.0) | 5 (0.8) | Multiple imputation |
| Number of painful regions | 0/730 (0.0) | 0 (0.0) | Complete |
| painDETECT-defined neuropathic-like pain | 15/730 (2.1) | 10 (1.6) | Multiple imputation |
| Pain Catastrophizing Scale | 18/730 (2.5) | 12 (1.9) | Multiple imputation |
| HADS anxiety score | 13/730 (1.8) | 9 (1.4) | Multiple imputation |
| HADS depression score | 14/730 (1.9) | 10 (1.6) | Multiple imputation |
| Kellgren–Lawrence grade | 0/730 (0.0) | 0 (0.0) | Complete |
| Preoperative alignment | 5/730 (0.7) | 4 (0.6) | Multiple imputation |
| Implant type | 0/730 (0.0) | 0 (0.0) | Complete |
| Patellar resurfacing | 0/730 (0.0) | 0 (0.0) | Complete |
| Surgical approach | 0/730 (0.0) | 0 (0.0) | Complete |
| Tourniquet use | 0/730 (0.0) | 0 (0.0) | Complete |
| Anesthesia type | 0/730 (0.0) | 0 (0.0) | Complete |
| Regional nerve block | 0/730 (0.0) | 0 (0.0) | Complete |
| Hospital length of stay | 0/730 (0.0) | 0 (0.0) | Complete |
| Any complication before the 3-month assessment | 0/730 (0.0) | 0 (0.0) | Complete |
| Pain-map acquisition method | 0/730 (0.0) | 0 (0.0) | Complete |
| Week-2 pain drawing | 21/730 (2.9) | 0 (0.0) | Not imputed |
| Week-6 pain drawing | 36/730 (4.9) | 0 (0.0) | Not imputed |
| Month-3 pain drawing | 25/730 (3.4) | 0 (0.0) | Not imputed |
| Week-2 knee NRS | — | 6/624 (1.0) | Multiple imputation |
| Week-6 knee NRS | — | 9/624 (1.4) | Descriptive/MI if modeled |
| Month-3 knee NRS | — | 8/624 (1.3) | Multiple imputation |
| Month-6 PEI | — | 27/624 (4.3) | Available-case summary |
| Month-12 PEI | — | 35/624 (5.6) | Available-case summary |
| Month-6 knee NRS | — | 14/624 (2.2) | Outcome not imputed |
| Month-12 persistent-pain outcome¹ | 35/659 (5.3) | 0/624 (0.0) | Outcome not imputed |
| Month-12 KOOS-JR | — | 17/624 (2.7) | Outcome not imputed |
| Month-12 Forgotten Joint Score-12 | — | 22/624 (3.5) | Outcome not imputed |
| Clinically important KOOS-JR improvement | — | 18/624 (2.9) | Outcome not imputed |
| Reoperation or revision status | — | 0/624 (0.0) | Complete |

¹The denominator is the 659 patients with an estimable early PEI slope.

| **Panel D. IPOW diagnostics and weighted association** | |
| --- | --- |
| **Diagnostic or estimate** | **Result** |
| Landmark target population | 730 |
| Early PEI slope estimable | 659/730 (90.3%) |
| Primary outcome observed among patients with estimable exposure | 624/659 (94.7%) |
| Overall analysis observation | 624/730 (85.5%) |
| Exposure-observation probability, median (IQR) | 0.92 (0.87–0.95) |
| Exposure-observation probability, range | 0.51–0.98 |
| Outcome-observation probability, median (IQR) | 0.96 (0.94–0.98) |
| Outcome-observation probability, range | 0.70–1.00 |
| Untruncated stabilized weight, mean (SD) | 1.003 (0.22) |
| Untruncated weight range | 0.88–2.08 |
| First and 99th weight percentiles | 0.89 and 1.74 |
| Truncated stabilized weight, mean (SD) | 0.998 (0.18) |
| Truncated weight range | 0.89–1.74 |
| Effective sample size | 604 |
| Maximum absolute SMD before weighting | 0.23 |
| Maximum absolute SMD after weighting | 0.06 |
| Unweighted adjusted OR per 1-SD slower contraction | 1.96 (1.51–2.54) |
| IPOW-adjusted OR per 1-SD slower contraction | 1.91 (1.46–2.50) |
| IPOW-adjusted P value | <0.001 |
