# Supplementary material for: Early spatial pain contraction and moderate-to-severe knee pain 12 months after total knee arthroplasty: a retrospective cohort study
Source: Front Pain Res (Lausanne). 2026 Oct 12;7:1948322. doi: 10.3389/fpain.2026.1948322 (PMC13696397; doi:10.3389/fpain.2026.1948322)
Supplement: Supplementary file 3 [file Table3.docx]

Supplementary Table 3. Quality control, reproducibility, and acquisition-method robustness of pain-drawing processing.

| **Panel A. Quality-control results for the primary exposure drawings** | | | |
| --- | --- | --- | --- |
| **Quality-control characteristic** | **n/N (%)** | | |
| Early postoperative drawings processed | 1,872/1,872 (100) | | |
| Paper-based drawings | 1,161/1,872 (62.0) | | |
| Electronic drawings | 711/1,872 (38.0) | | |
| Passed initial quality control without correction | 1,833/1,872 (97.9) | | |
| Required secondary review or correction | 39/1,872 (2.1) | | |
| Residual annotation or nonpain marking | 18/1,872 (1.0) | | |
| Landmark-placement or registration error | 12/1,872 (0.6) | | |
| Scanning, background, or cropping artifact | 9/1,872 (0.5) | | |
| Successfully resolved by consensus | 39/39 (100) | | |
| Excluded after quality-control review | 0/1,872 (0.0) | | |
| **Panel B. Inter- and intra-rater reproducibility** | | | |
| **Measure** | **Inter-rater result** | **Intra-rater result** | |
| Patients included | 125 | 125 | |
| Drawings included | 375 | 375 | |
| Paper-based drawings | 234 | 234 | |
| Electronic drawings | 141 | 141 | |
| PEI absolute-agreement ICC (95% CI), all drawings | 0.987 (0.983–0.990) | 0.994 (0.992–0.996) | |
| PEI ICC (95% CI), paper drawings | 0.985 (0.980–0.989) | 0.993 (0.990–0.995) | |
| PEI ICC (95% CI), electronic drawings | 0.991 (0.987–0.994) | 0.996 (0.994–0.997) | |
| Mean absolute PEI difference, percentage points | 0.24 | 0.14 | |
| Mean PEI difference, percentage points | 0.03 | 0.01 | |
| Bland–Altman 95% limits of agreement, percentage points | −0.59 to 0.65 | −0.37 to 0.39 | |
| Median Dice coefficient (IQR), all drawings | 0.949 (0.927–0.968) | 0.971 (0.956–0.983) | |
| Median Dice coefficient (IQR), paper drawings | 0.943 (0.919–0.965) | 0.968 (0.951–0.981) | |
| Median Dice coefficient (IQR), electronic drawings | 0.958 (0.939–0.974) | 0.976 (0.963–0.986) | |
| Early PEI-slope ICC (95% CI) | 0.982 (0.974–0.988) | 0.991 (0.987–0.994) | |
| Mean absolute slope difference, log(PEI+1) units/week | 0.0042 | 0.0028 | |
| Mean slope difference, log(PEI+1) units/week | 0.0004 | 0.0002 | |
| Slope 95% limits of agreement, log(PEI+1) units/week | −0.0100 to 0.0108 | −0.0070 to 0.0074 | |
| **Panel C. Robustness according to pain-drawing acquisition method** | | | |
| **Analysis** | **Paper-based** | **Electronic** | **Overall or interaction result** |
| Patients, n | 387 | 237 | 624 |
| Twelve-month pain events, n (%) | 56 (14.5) | 36 (15.2) | 92 (14.7) |
| Adjusted OR per 1-SD slower contraction (95% CI) | 1.91 (1.39–2.63) | 2.04 (1.34–3.12) | 1.95 (1.50–2.53) |
| P value for slope association | <0.001 | 0.001 | <0.001 |
| Slope-by-acquisition-method interaction | — | — | P=0.79 |

PEI agreement was evaluated using two-way absolute-agreement intraclass correlation coefficients. Intra-rater processing was repeated after at least four weeks. The Dice coefficient was calculated as twice the intersection of the two pain-positive masks divided by their combined pain-positive area.

Acquisition-method models included the Model 2 baseline and perioperative covariates. The overall estimate additionally adjusted for acquisition method; the interaction model included the main effects of the standardized PEI slope and acquisition method and their interaction.
