# Supplementary material for: Early spatial pain contraction and moderate-to-severe knee pain 12 months after total knee arthroplasty: a retrospective cohort study
Source: Front Pain Res (Lausanne). 2026 Oct 12;7:1948322. doi: 10.3389/fpain.2026.1948322 (PMC13696397; doi:10.3389/fpain.2026.1948322)
Supplement: Supplementary file 4 [file Table4.docx]

Supplementary Table 4. Estimation of patient-specific Pain Extent Index slopes, model diagnostics, and sensitivity analyses.

| **Panel A. Linear mixed-effects model used to estimate early patient-specific PEI slopes** | | | | |
| --- | --- | --- | --- | --- |
| **Domain** | **Item** | **Specification or result** | | |
| Data | Participants | 624 patients | | |
| Data | Longitudinal observations | 1,872 observations; three postoperative measurements per patient | | |
| Data | Assessment times | Postoperative week 2, week 6, and month 3; exact elapsed weeks were used, with time centered at week 2 | | |
| Model | Outcome | log(whole-body PEI + 1) | | |
| Model | Fixed effects | Intercept and linear time | | |
| Model | Random effects | Patient-specific intercept and linear time slope with an unstructured 2 × 2 covariance matrix | | |
| Estimation | Software and estimator | R version 4.4.2 using lme4 version 1.1-35.5; restricted maximum likelihood | | |
| Fixed effect | Population-average time slope | −0.067 log(PEI + 1) units/week (95% CI, −0.070 to −0.064; P<0.001) | | |
| Variance | Random-intercept variance | 0.276 (SD, 0.525) | | |
| Variance | Random-slope variance | 0.0021 (SD, 0.046 log[PEI + 1] units/week) | | |
| Variance | Intercept-slope covariance | Approximately −0.010, calculated from the rounded SDs and correlation | | |
| Variance | Intercept-slope correlation | −0.42 | | |
| Variance | Residual variance | 0.041 (SD, 0.202) | | |
| Slope output | Empirical Bayes slope distribution | Median, −0.065 (IQR, −0.090 to −0.043) log(PEI + 1) units/week; SD, 0.039 | | |
| Shrinkage | Empirical Bayes versus latent slope spread | Empirical Bayes slope SD, 0.039; estimated latent random-slope SD, 0.046; variance ratio, 0.72 | | |
| Shrinkage | Conditional slope reliability | Median, 0.75 (IQR, 0.70–0.79) | | |
| Convergence | Optimizer and numerical convergence | bobyqa optimizer; convergence code 0; no optimizer warnings; maximum absolute scaled gradient, 0.00031 | | |
| Convergence | Hessian and singular-fit assessment | Positive-definite Hessian (condition number, 18.6); fit was not singular at a tolerance of 1 × 10⁻⁴ | | |
| Diagnostics | Residual diagnostics | Standardized conditional residuals had a mean of 0.001 and SD of 0.998. Residual-versus-fitted and Q-Q plots showed no important heteroscedasticity or departure from normality. Maximum participant-level Cook’s distance was 0.18 | | |
| **Panel B. Shared-parameter joint model accounting for latent-slope uncertainty** | | | | |
| **Domain** | **Item** | **Specification or result** | | |
| Framework | Joint likelihood | The longitudinal PEI measurements and binary 12-month moderate-to-severe knee-pain outcome were fitted simultaneously, with both submodels linked through the same patient-level random effects | | |
| Longitudinal submodel | Response distribution | Normal model for yit=log(PEIit+1) | | |
| Longitudinal submodel | Linear predictor | yit=β0+β1tit+b0i+b1itit+εit, where time was expressed in exact weeks and centered at week 2 | | |
| Longitudinal submodel | Random effects | (b0i,b1i)′ followed a bivariate normal distribution with an unstructured covariance matrix; εit followed a normal distribution with variance σ2 | | |
| Outcome submodel | Distribution and link | Yi followed a Bernoulli distribution with a logit link, where Yi=1 denoted symptom-defined moderate-to-severe knee pain at 12 months | | |
| Outcome submodel | Linear predictor | logit{Pr(Yi=1)}=γ0+Xi′γ+ψzi | | |
| Shared parameter | Latent slope term | zi was the posterior latent patient-specific slope (β1+b1i), centered and divided by its posterior SD; positive values represented slower contraction | | |
| Adjustment | Covariates | The same prespecified baseline and perioperative covariates as primary Model 2 were included in Xi | | |
| Estimand | Association parameter | exp(ψ): posterior OR for moderate-to-severe knee pain at 12 months per 1-SD slower latent spatial contraction | | |
| Bayesian specification | Prior distributions | Normal(0, 2.5) for longitudinal fixed effects; Normal(0, 1.5) for standardized logit-scale covariate coefficients; Normal(0, 1) for ψ; half-Student-t(3, 0, 2.5) for SD parameters; and LKJ(2) for the random-effects correlation matrix | | |
| Computation | Software and sampler | R version 4.4.2 with cmdstanr version 0.8.1 and CmdStan version 2.36.0; No-U-Turn Hamiltonian Monte Carlo sampler | | |
| Computation | MCMC settings | Four chains with 6,000 iterations per chain, including 3,000 warm-up iterations; 12,000 retained draws; adapt_delta=0.99; max_treedepth=15; random seed=20260418 | | |
| Convergence | R-hat | R-hat ranged from 1.000 to 1.004 for all monitored parameters; maximum R-hat, 1.004 | | |
| Convergence | Effective sample size | Minimum bulk effective sample size, 2,486; minimum tail effective sample size, 2,057. For ψ, bulk and tail effective sample sizes were 6,842 and 5,911, respectively | | |
| Convergence | Sampler diagnostics | No divergent transitions or maximum-treedepth exceedances occurred. Chain-specific energy Bayesian fraction of missing information ranged from 0.89 to 1.03 | | |
| Model checking | Posterior predictive assessment | The posterior-replicated outcome rate was 14.8% (95% posterior predictive interval, 11.9%–17.9%), compared with the observed rate of 14.7%. Replicated longitudinal means and SDs covered the observed values at all three assessments; Bayesian predictive P values ranged from 0.46 to 0.55 | | |
| Association | Joint-model estimate | Adjusted posterior OR, 2.05 (95% CrI, 1.54–2.73) per 1-SD slower latent contraction; similar in direction and magnitude to the primary two-stage estimate | | |
| **Panel C. Primary and sensitivity analyses of the association with moderate-to-severe knee pain at 12 months** | | | | |
| **Analysis** | **Patients/outcome events** | **Exposure contrast** | **Adjusted effect (95% interval)** | **P value** |
| Primary two-stage PEI-slope analysis | 624/92 | Per 1-SD slower contraction | OR, 1.96 (95% CI, 1.51–2.54) | <0.001 |
| Excluding patients with an adjudicated specific alternative explanation for pain | 612/80 | Per 1-SD slower contraction | OR, 1.89 (95% CI, 1.42–2.52) | <0.001 |
| Absolute PEI change | 624/92 | Per 1-SD less contraction | OR, 1.82 (95% CI, 1.45–2.29) | <0.001 |
| Relative spatial pain-contraction rate | 624/92 | Per 1-SD lower rate | OR, 1.74 (95% CI, 1.38–2.19) | <0.001 |
| Time-weighted PEI area under the curve | 624/92 | Per 1-SD higher AUC | OR, 1.89 (95% CI, 1.49–2.39) | <0.001 |
| Operated lower-extremity PEI slope | 624/92 | Per 1-SD slower contraction | OR, 1.71 (95% CI, 1.36–2.15) | <0.001 |
| Operated-knee PEI slope | 624/92 | Per 1-SD slower contraction | OR, 1.65 (95% CI, 1.31–2.08) | <0.001 |
| Intensity-weighted pain-area slope | 624/92 | Per 1-SD slower contraction | OR, 1.84 (95% CI, 1.45–2.34) | <0.001 |
| Shared-parameter joint model | 624/92 | Per 1-SD slower latent contraction | Posterior OR, 2.05 (95% CrI, 1.54–2.73) | NA |
| Stabilized inverse-probability-of-observation weighting | 624/92¹ | Per 1-SD slower contraction | OR, 1.91 (95% CI, 1.46–2.50) | <0.001 |
| **Panel D. Adjudicated specific explanations among patients meeting the 12-month knee-pain outcome** | | | | |
| **Principal adjudicated explanation** | **Patients, n** | **Percentage of 92 outcome events** | | |
| Symptomatic instability | 4 | 4.3 | | |
| Arthrofibrosis or clinically important stiffness | 3 | 3.3 | | |
| Patellofemoral or extensor-mechanism complication | 2 | 2.2 | | |
| Component malposition or malalignment | 1 | 1.1 | | |
| Aseptic loosening | 1 | 1.1 | | |
| Periprosthetic joint infection | 1 | 1.1 | | |
| **Any adjudicated specific alternative explanation** | **12** | **13** | | |

Footnotes

a Empirical Bayes slopes are partially pooled estimates. Conditional slope reliability was calculated for each patient.

The reported median and IQR were calculated across patients.

b Frequentist adjusted models and the binary submodel of the joint analysis included age, sex, body mass index, Charlson Comorbidity Index, chronic low back pain, fibromyalgia or another chronic widespread pain condition, preoperative opioid use, preoperative operated-knee NRS, preoperative whole-body PEI, preoperative KOOS-JR, painDETECT-defined neuropathic-like pain, Pain Catastrophizing Scale score, Hospital Anxiety and Depression Scale anxiety and depression scores, and any complication before the 3-month assessment.

c The exclusion sensitivity analysis used the same exposure scaling and covariate specification as the primary two-stage model. The 12 affected patients were excluded, leaving 612 patients and 80 outcome events.

d A specific alternative explanation was adjudicated using the clinical history, physical examination, postoperative radiographs, and, when clinically indicated, inflammatory markers, joint aspiration, stress radiographs, or computed tomography. Prespecified causes included periprosthetic joint infection; aseptic loosening, subsidence, or osteolysis; periprosthetic fracture; symptomatic instability; clinically important component malposition or malalignment; patellofemoral or extensor-mechanism complications; arthrofibrosis with clinically important stiffness; and another documented musculoskeletal or neurologic condition judged to account for the operated-knee pain.

e Categories in Panel D represent the principal adjudicated explanation and are mutually exclusive. The presence of an adjudicated explanation did not necessarily result in reoperation or revision.

f The effective sample size in the inverse-probability-of-observation-weighted analysis was 604.

g CI denotes a frequentist confidence interval, whereas CrI denotes a Bayesian credible interval. A Bayesian P value was not reported for the shared-parameter joint model.

Abbreviations: AUC, area under the curve; CI, confidence interval; CrI, credible interval; IQR, interquartile range; KOOS-JR, Knee injury and Osteoarthritis Outcome Score for Joint Replacement; MCMC, Markov chain Monte Carlo; NRS, numerical rating scale; OR, odds ratio; PEI, Pain Extent Index; SD, standard deviation.
